# Supplementary material for: Unveiling the Bioactive Potential of the Invasive Jellyfish Phyllorhiza punctata Through Integrative Transcriptomic and Proteomic Analyses
Source: Biomolecules. 2025 Aug 4;15(8):1121. doi: 10.3390/biom15081121 (PMC12383608; doi:10.3390/biom15081121)
Supplement: Supplementary file 1 [file biomolecules-15-01121-s001.zip › Suplementary Material/FileS3/PEPFOLD4/AKU77028_10750/clusters.html]

 PEP-FOLD clusters


Download archive of all models  here   

|  |  |  |
| --- | --- | --- |
| # | file | sOPEP |
| # cluster 1 - Size: 71 |  |  |
|  | AKU77028\_10750-model1 | -20.8097 |
|  | AKU77028\_10750-model1.1 | -20.7321 |
|  | AKU77028\_10750-model1.2 | -20.6647 |
|  | AKU77028\_10750-model1.3 | -20.626 |
|  | AKU77028\_10750-model1.4 | -20.623 |
|  | AKU77028\_10750-model1.5 | -20.623 |
|  | AKU77028\_10750-model1.6 | -20.623 |
|  | AKU77028\_10750-model1.7 | -20.6016 |
|  | AKU77028\_10750-model1.8 | -20.6016 |
|  | AKU77028\_10750-model1.9 | -20.6016 |
|  | AKU77028\_10750-model1.10 | -20.6002 |
|  | AKU77028\_10750-model1.11 | -20.5925 |
|  | AKU77028\_10750-model1.12 | -20.5851 |
|  | AKU77028\_10750-model1.13 | -20.5733 |
|  | AKU77028\_10750-model1.14 | -20.5617 |
|  | AKU77028\_10750-model1.15 | -20.5617 |
|  | AKU77028\_10750-model1.16 | -20.5599 |
|  | AKU77028\_10750-model1.17 | -20.5599 |
|  | AKU77028\_10750-model1.18 | -20.5593 |
|  | AKU77028\_10750-model1.19 | -20.5593 |
|  | AKU77028\_10750-model1.20 | -20.5404 |
|  | AKU77028\_10750-model1.21 | -20.5404 |
|  | AKU77028\_10750-model1.22 | -20.5404 |
|  | AKU77028\_10750-model1.23 | -20.5404 |
|  | AKU77028\_10750-model1.24 | -20.5404 |
|  | AKU77028\_10750-model1.25 | -20.5404 |
|  | AKU77028\_10750-model1.26 | -20.5356 |
|  | AKU77028\_10750-model1.27 | -20.5356 |
|  | AKU77028\_10750-model1.28 | -20.5356 |
|  | AKU77028\_10750-model1.29 | -20.5274 |
|  | AKU77028\_10750-model1.30 | -20.5152 |
|  | AKU77028\_10750-model1.31 | -20.5152 |
|  | AKU77028\_10750-model1.32 | -20.5059 |
|  | AKU77028\_10750-model1.33 | -20.5059 |
|  | AKU77028\_10750-model1.34 | -20.4835 |
|  | AKU77028\_10750-model1.35 | -20.4835 |
|  | AKU77028\_10750-model1.36 | -20.4773 |
|  | AKU77028\_10750-model1.37 | -20.4557 |
|  | AKU77028\_10750-model1.38 | -20.4555 |
|  | AKU77028\_10750-model1.39 | -20.4412 |
|  | AKU77028\_10750-model1.40 | -20.4293 |
|  | AKU77028\_10750-model1.41 | -20.4187 |
|  | AKU77028\_10750-model1.42 | -20.414 |
|  | AKU77028\_10750-model1.43 | -20.394 |
|  | AKU77028\_10750-model1.44 | -20.3881 |
|  | AKU77028\_10750-model1.45 | -20.3881 |
|  | AKU77028\_10750-model1.46 | -20.3871 |
|  | AKU77028\_10750-model1.47 | -20.3829 |
|  | AKU77028\_10750-model1.48 | -20.374 |
|  | AKU77028\_10750-model1.49 | -20.3587 |
|  | AKU77028\_10750-model1.50 | -20.3546 |
|  | AKU77028\_10750-model1.51 | -20.3472 |
|  | AKU77028\_10750-model1.52 | -20.3298 |
|  | AKU77028\_10750-model1.53 | -20.3022 |
|  | AKU77028\_10750-model1.54 | -20.2864 |
|  | AKU77028\_10750-model1.55 | -20.2797 |
|  | AKU77028\_10750-model1.56 | -20.2758 |
|  | AKU77028\_10750-model1.57 | -20.2614 |
|  | AKU77028\_10750-model1.58 | -20.2537 |
|  | AKU77028\_10750-model1.59 | -20.2528 |
|  | AKU77028\_10750-model1.60 | -20.252 |
|  | AKU77028\_10750-model1.61 | -20.2405 |
|  | AKU77028\_10750-model1.62 | -20.2322 |
|  | AKU77028\_10750-model1.63 | -20.2322 |
|  | AKU77028\_10750-model1.64 | -20.227 |
|  | AKU77028\_10750-model1.65 | -20.218 |
|  | AKU77028\_10750-model1.66 | -20.2066 |
|  | AKU77028\_10750-model1.67 | -20.1926 |
|  | AKU77028\_10750-model1.68 | -20.1701 |
|  | AKU77028\_10750-model1.69 | -20.0608 |
|  | AKU77028\_10750-model1.70 | -19.9947 |
| # cluster 2 - Size: 29 |  |  |
|  | AKU77028\_10750-model2 | -20.5869 |
|  | AKU77028\_10750-model2.1 | -20.4963 |
|  | AKU77028\_10750-model2.2 | -20.4963 |
|  | AKU77028\_10750-model2.3 | -20.4963 |
|  | AKU77028\_10750-model2.4 | -20.4963 |
|  | AKU77028\_10750-model2.5 | -20.4945 |
|  | AKU77028\_10750-model2.6 | -20.4257 |
|  | AKU77028\_10750-model2.7 | -20.4257 |
|  | AKU77028\_10750-model2.8 | -20.4257 |
|  | AKU77028\_10750-model2.9 | -20.4164 |
|  | AKU77028\_10750-model2.10 | -20.4065 |
|  | AKU77028\_10750-model2.11 | -20.3987 |
|  | AKU77028\_10750-model2.12 | -20.3987 |
|  | AKU77028\_10750-model2.13 | -20.3987 |
|  | AKU77028\_10750-model2.14 | -20.3966 |
|  | AKU77028\_10750-model2.15 | -20.3884 |
|  | AKU77028\_10750-model2.16 | -20.3826 |
|  | AKU77028\_10750-model2.17 | -20.3642 |
|  | AKU77028\_10750-model2.18 | -20.3532 |
|  | AKU77028\_10750-model2.19 | -20.3295 |
|  | AKU77028\_10750-model2.20 | -20.3269 |
|  | AKU77028\_10750-model2.21 | -20.3119 |
|  | AKU77028\_10750-model2.22 | -20.2596 |
|  | AKU77028\_10750-model2.23 | -20.257 |
|  | AKU77028\_10750-model2.24 | -20.2059 |
|  | AKU77028\_10750-model2.25 | -20.2033 |
|  | AKU77028\_10750-model2.26 | -20.1843 |
|  | AKU77028\_10750-model2.27 | -20.1662 |
|  | AKU77028\_10750-model2.28 | -20.0401 |
|  |  |  |
